# Standardised Ethanol Extract of *Tithonia diversifolia* (Hemsley) A Gray Leaves Improve Insulin Sensitivity and Increase Mitochondrial DNA Copy Numbers in Skeletal Muscles of Streptozotocin-Nicotinamide-Induced Rats

**DOI:** 10.21315/mjms2022.29.3.5

**Published:** 2022-06-28

**Authors:** Rochmy Istikharah, Dwi Aris Agung Nugrahaningsih, Ahmad Hamim Sadewa, Mae Sri Hartati Wahyuningsih

**Affiliations:** 1 Department of Pharmacy, Universitas Islam Indonesia, Yogyakarta, Indonesia; 2 Doctoral candidate, Faculty of Medicine, Public Health and Nursing, Universitas Gadjah Mada, Yogyakarta, Indonesia; 3 Department of Pharmacology and Therapy, Faculty of Medicine, Public Health and Nursing, Universitas Gadjah Mada, Yogyakarta, Indonesia; 4 Center of Herbal Medicine, Faculty of Medicine, Public Health and Nursing, Universitas Gadjah Mada, Yogyakarta, Indonesia; 5 Department of Biochemistry, Faculty of Medicine, Public Health and Nursing, Universitas Gadjah Mada, Yogyakarta, Indonesia

**Keywords:** Tithonia, diabetes mellitus, experimental, hypoglycemic agents, insulin resistance, DNA, mitochondrial

## Abstract

**Background:**

In this study, we examined the anti-diabetic activity of standardised extracts of *Tithonia diversifolia* (Hemsley) A Gray (*T. diversifolia*) leaves for their effects on insulin resistance and mitochondrial DNA (mtDNA) copy number.

**Methods:**

*T. diversifolia* leaves were extracted using an ultrasound-assisted method and standardised using Tagitinin C. There were six groups: i) normal control; ii) diabetic group; iii) metformin group (300 mg/kg) and iv) groups treated with three different doses of extract (50 mg/kg, 100 mg/kg and 150 mg/kg). Blood samples were taken before and after 28 days of treatment for fasting plasma glucose (FPG) and insulin analysis, which were used for a Homeostatic Model Assessment for Insulin Resistance (HOMA-IR) calculation. The soleus and gastrocnemius muscles were harvested after 28 days of treatment for the measurement of mtDNA copy number.

**Results:**

The results showed an improvement in blood glucose levels and HOMA-IR scores in all treatment groups. The results of mtDNA copy number analysis also revealed significant improvement with the highest number observed at an extract dose of 100 mg/kg in which the mtDNA copy number increased up to 3 times in the soleus muscles (*P* < 0.001).

**Conclusion:**

*T. diversifolia* extract has the potential to be used as an anti-diabetic agent that improves insulin resistance, possibly by increasing mtDNA content.

## Introduction

Insulin resistance is an abnormal condition that occurs in type 2 diabetic patients. A previous study showed that disruption of insulin secretion by pancreatic beta cells generally occurs because of insulin resistance in skeletal muscle, liver, and adipose tissues (1). However, insulin resistance in the muscle tissue is considered an initial disorder or the main dysfunction causing hyperglycaemic events, which ultimately decrease β-cell function. This occurs because skeletal muscle is a predominant target for glucose uptake mediated by insulin, especially during postprandial conditions (2).

In skeletal muscles, several conditions may contribute to insulin resistance, including disorders of the insulin signal, glucose transport, glucose phosphorylation, glycogen synthesis and the oxidative activity of mitochondria (3). Also, mitochondrial dysfunction is believed to be involved in the mechanism of insulin resistance (4). Mitochondrial dysfunction can be determined as a change in the size or number of mitochondria, a change in the number of mitochondrial proteins, or a decrease in the activity of enzymes that are important to mitochondrial oxidation (5). Nevertheless, the relationship between the incidence of mitochondrial dysfunction and type 2 diabetes mellitus (T2DM) is unclear. In other words, it may be the cause or the result of insulin resistance that contributes to T2DM.

*Tithonia diversifolia* (Hemsley) A Gray extract antihyperglycaemic activity that is beneficial for the treatment of T2DM (6). In vitro studies have demonstrated the ability of *T. diversifolia* to act as a peroxisome proliferator activator receptor (PPARα/γ) agonist (7), which increases 5’AMP-activated protein kinase (AMPK) phosphorylation (8). The PPARα/γ agonist and AMPK are positively correlated with increased peroxisome proliferator-activated receptor-γ coactivator-1α (PGC1α) expression, which plays a role in mitochondrial biogenesis (9). The initial parameter used to identify an increase in mitochondrial biogenesis is mitochondrial DNA (mtDNA) copy number. Changes in mtDNA copy number can protect cells from mitochondrial damage resulting from oxidative stress during the development of insulin resistance (10). Also, previous studies have demonstrated the antioxidant activities of *T. diversifolia* (11–14). Therefore, we evaluated the activity of *T. diversifolia* standardised extract as an anti-diabetic through a mechanism of increased insulin sensitivity and mtDNA copy number.

## Methods

### T. diversifolia Extract Preparation

*T. diversifolia* leaves were purchased from Merapi Farma Ltd., Yogyakarta, Indonesia and were authenticated at the Laboratory of Pharmaceutical Biology, Department of Pharmacy, Universitas Islam Indonesia with reference number 01/UII/JurFar/det/VIII/2018. The extract was prepared from the dried and powdered leaves of *T. diversifolia* by ultrasound-assisted extraction at 20 kHz and a temperature of 40 °C for 30 min. The solution was then filtered with a Buchner funnel and prepared into a thick extract using a rotary evaporator (Heidolph-L4000, Germany).

### Extract Standardisation

Extract standardisation was done following the regulations of the National Agency of Drug and Food Control, Indonesia with respect to the safety and quality requirements of traditional medicines on specific and non-specific parameters. The examination of specific parameters included the qualitative identification of compounds contained in the extract and the quantitative measurement of Tagitinin C as a marker. The inspection included non-specific parameters, such as water content, total ash, residual solvent, microbial contamination and heavy metal analysis.

Qualitative tests of extract content were performed to identify flavonoids, alkaloids, saponins, terpenoids and polyphenols. Identification of Tagitinin C as a marker compound in extracts was done by thin layer chromatography with a benzene:ethyl acetate (2:1 v/v) mobile phase as previously described, followed by quantitative analysis with a densitometer (CAMAG TLC Scanner 3, Swiss).

The non-specific parameters tested included water content and total ash by a gravimetric assay, lead (Pb) and cadmium (Cd) by atomic absorption spectrometry (Perkin Elmer PinnAAcle 900T, USA) and residual ethanol solvent by gas chromatography/mass spectrometry (Shimadzu QP2010 SE, Japan). Microbial contamination was evaluated by total plate counts using plate count agar, yeast mold numbers with Czapek Dox Agar and identification of various microbes including *Staphylococcus aureus*, *Escherichia coli*, *Pseudomonas spp*. and *Salmonella spp*.

### Animals and Experimental Design

This research was approved by the Medical and Health Research Ethics Committee, Integrated Research and Testing Laboratory, Universitas Gadjah Mada. The study used 30 six-week-old Wistar male rats weighing 170 g–240 g obtained from the Department of Pharmacology and Therapy, Faculty of Medicine, Public Health and Nursing, Universitas Gadjah Mada. Rats were acclimatised 7 days before the study was conducted. The rats were housed in groups of 3 in a standard cage measuring 48 cm × 35 cm × 22 cm at room temperature (25 ± 2 °C) and a 12 h dark/light cycle. Rats had free access to food and water.

The rats were first fasted for 8 h, then blood was drawn to determine fasting plasma glucose (FPG) and plasma insulin levels at baseline conditions. Twenty-five rats were subject to diabetes induction, whereas five rats were not induced (normal control group). Diabetes induction was done by injecting rats with 230 mg/kg intraperitoneal nicotinamide (Sigma-Aldrich, Singapore) dissolved in 0.9% NaCl. Fifteen minutes later, the rats were injected intraperitoneally with 65 mg/kg streptozotocin (BioWorld, USA) which was dissolved in cold citrate buffer (pH 4.5). Three weeks later, the rats were fasted for 8 h and their blood was drawn to measure plasma glucose levels to determine the success of diabetes induction. The rats were considered diabetic and included in the study if FPG levels > 150 mg/dL. They were subsequently divided into the following six groups:

- Group 1: Normal control
- Group 2: Untreated diabetic
- Group 3: Diabetic + 300 mg/kg metformin p.o.
- Group 4: Diabetic + 50 mg/kg extracts p.o.
- Group 5: Diabetic + 100 mg/kg extracts p.o.
- Group 6: Diabetic + 150 mg/kg extracts p.o.

The treatments either with metformin or extract were carried out for 28 days. During the experimental period, blood sampling was performed three times as follows: before the experiment (baseline condition of FPG and insulin), 3 weeks after streptozotocin-nicotinamide diabetic induction (FPG after induction) and 28 days after treatment (FPG and insulin after treatment). At the end of the experiment, the rats were euthanised following blood collection. Soleus and gastrocnemius skeletal muscles were excised and stored at 80 °C until analysis.

### Specimen Collection and Euthanasia

All blood specimen collections were performed by the following procedure. After rats had fasted overnight, they were anaesthetised intraperitoneally with 0.15 mL/100 g body weight ketamine-xylazine combination containing 100 mg/kg ketamine and 10 mg/kg xylazine. Blood was drawn from the anaesthetised-rats through the retro-orbital veins into heparinised-capillary tubes. Blood was collected in a microtube containing ethylenediaminetetraacetic acid (EDTA). At the end of the study, all animals were euthanised using three times the anaesthetic dose (0.45 mL/100 g body weight ketamine-xylazine combination).

### Biochemical Analysis

Plasma separation was done within 60 min following blood collection by centrifugation at 3000 rpm for 15 min. Plasma was separated and collected into sterile microtubes, then used for glucose analysis and stored at −20 °C until further analysis. Glucose measurements on blood samples at day 0 (baseline), induction and termination were done in duplicate with the GOD-PAP method according to the manufacturer’s protocol (Glucose GOD FS, DiaSys, Germany). Internal quality control yielded 1.5% precision.

### Insulin Resistance Analysis

Fasting plasma insulin was measured on blood specimens taken on day 0 and the last day of treatment. Each sample was tested in duplicate using an Enzyme-linked Immunosorbent Assay method according to the manufacturer’s protocol (FineTest, Wuhan Fine Biotech Co. Ltd, China). The Homeostatic Model Assessment for Insulin Resistance (HOMA-IR) score was calculated as fasting plasma insulin (μU/mL) × FPG (mmol/L)/correction factor. The correction factor was calculated by multiplying the median glucose value with the median insulin value at baseline conditions (15). The baseline for FPG ranged from 4.77 mmol/L to 6.68 mmol/L (median 5.92 mmol/L) and plasma insulin concentrations ranged from 0.85 μU/mL to 3.65 μU/mL (median 2.54), thus the correction factor was 15.06.

### Mitochondria DNA Copy Number Analysis

Total DNA was isolated from the soleus and gastrocnemius tissues following the manufacturer’s protocol (FavorPrep Tissue Genomic DNA Extraction Kit, Favorgen, Taiwan). The concentration and quality of the isolated DNA was determined (MaestroNano Pro MN-913A, Maestrogen, Taiwan). The mtDNA copy number was analysed using quantitative real-time PCR (BioRad CFX96, USA). NADH dehydrogenase subunit 1 (ND1) was used as a mitochondrial gene, whereas β-actin was used as a nuclear gene. The primers used for the study were designed using the Primer Blast program and the appropriate database (Table 1). Quantitative real-time PCR analysis was performed using a DNA concentration of 50 ng, 5 pmol primers, 5 μL of real-time master mix (ExcelTaq 2x Fast Q-PCR Master Mix, Smobio, Taiwan) and nuclease-free water to a final volume of 10 μL. The amplification protocol was as follows: pre-denaturation at 95 °C for 2 min, 38 cycles at 95 °C for 15 s, 60 °C for 1 min, followed by a melt curve analysis from 65 °C to 95 °C. The mtDNA copy number was calculated using the 2^−ΔΔCT^ method as previously described (16).

### Statistical Analysis

Data are presented as mean (standard deviation [SD]). Data normality was assessed with the Shapiro-Wilk test. Differences between groups were tested with a one-way analysis of variance (ANOVA) followed by LSD post-hoc multiple comparisons test for parametric data and a Mann-Whitney U test for non-parametric data. *P*-values less than 0.05 were considered statistically significant. All statistical analyses were performed using IBM SPSS Statistics Software (version 21).

## Results

### Standardised Extract of T. diversifolia as an Anti-Diabetic Agent

*T. diversifolia* leaves were extracted by an ultrasound-assisted method to produce a 10.71% w/w solution with a profile shown in Table 2. Identification of the specific parameters showed that the *T. diversifolia* extract contained flavonoids, alkaloids, saponins and polyphenols. Also, quantitative analysis of Tagitinin C as a biomarker for *T. diversifolia* using thin layer chromatography (TLC)-densitometry resulted in a value of 7.60% (w/w). These results were supported by non-specific parameters which indicated that the extract was free of microbial and heavy metal contamination. In general, the extract was standardised as good quality.

### Development of Type 2 Diabetic Rats

Before the experiment, all rats were in the normal range of FPG, insulin and HOMA-IR score (Table 3). We used a combination of streptozotocin and nicotinamide to induce T2DM based on previously described methods (17–18). The FPG levels were examined 3 weeks after induction and the rats were identified as diabetic if FPG was greater than 150 mg/dL (Table 3). This experiment showed that the rats still had diabetes until 7 weeks after induction (Table 4, diabetic group), whereas the overall diabetic induction success rate was 80%, whereas the remainder exhibited mortality (6%), non-diabetic phenotype (10%), or very high FPG (4%) (> 400 mg/dL).

The success of inducing diabetes mellitus (DM) was also evident by the insulin profile of the rats (Table 4, diabetic group), which did not exhibit hypoinsulinemia, which usually occurs from a single STZ administration, but showed a significantly different form of hyperinsulinemia (3.79 ± 0.49 μU/mL) compared with the normal group (2.55 ± 0.55 μU/mL; *P* < 0.001). The HOMA-IR (Table 4) in the diabetic group ranged from 2.60 to 3.76, whereas it was considered resistant if HOMA-IR > 2.6 (19). These results show that this animal model represents the conditions of type 2 DM observed in humans.

### T. diversifolia Attenuates Insulin Sensitivity

The blood glucose profile showed that after 28 days of treatment, there was a significant decrease in all groups (*P* < 0.001) compared with the diabetic group. The FPG level returned to normal levels in all treated rats, either with metformin or extract treatment (Table 4). Moreover, the insulin profile showed no significant difference compared with the normal group in all extract-treated groups (Table 4). Thus, the HOMA-IR score (Table 4) showed that all treatment groups have a score that differed significantly from the diabetic group (*P* = 0.009) but did not differ significantly from the normal group (*P* > 0.05).

### T. diversifolia Increases mtDNA Content

The effect of *T. diversifolia* on mtDNA copy number in diabetic rats was determined using soleus and gastrocnemius muscle mitochondrial numbers. The results of mtDNA analysis (Table 5) indicated that diabetic condition caused a significant decrease in the amount of mtDNA in the soleus muscle (*P* = 0.004). *T. diversifolia* extract at doses of 100 mg/kg and 150 mg/kg showed a significantly increased the mtDNA ratio in the soleus muscle compared with the diabetic group (*P* < 0.001). The activity of the 100 mg/kg and 150 mg/kg dose was also significantly higher than that of metformin at 300 mg/kg (*P*-value < 0.001 and 0.003, respectively). Meanwhile, there was an approximate 25% decrease in the gastrocnemius muscle in diabetic rats, although it was not significantly different from the normal group (*P* = 0.878). Metformin and extract activity in the gastrocnemius muscle was also significantly increased compared with the diabetic group (*P* < 0.05) and was not significantly different from the normal group.

## Discussion

In our previous study, *T. diversifolia* extract was shown to exert anti-hyperglycaemic effects (6). Therefore, in the present study, we improved the quality and reproducibility of the extract through a more complete standardisation process that included several specific and non-specific parameters. In addition, its activity in overcoming insulin resistance in T2DM was assessed. Using an ultrasound-assisted extraction method, we produced a high-yield extract within a short time (30 min). The identification of specific parameters revealed that the extract contained three times higher levels of Tagitinin C compared with that achieved from the previous conventional maceration method (20). Silva et al. (21) also reported that ultrasound-assisted extraction was superior to dynamic maceration for obtaining Tagitinin C. Our results are consistent with that of previous studies showing that *T. diversifolia* extracts contained flavonoids, alkaloids, saponins and polyphenols (13, 22).

In the present study, we used the HOMA-IR score to determine the occurrence of insulin resistance. This calculation was done by adjusting the correction factor using the animal conditions at baseline. This approach is consistent with the idea that HOMA-IR in humans cannot be used in animals without making adjustments (23–24). The animals in the diabetic group exhibited insulin resistance with a HOMA-IR score higher than 2.6 which lasted up to 7 weeks following induction. We conclude that this model is compatible with T2DM in humans. Therefore, the STZ-NA combination induction method applied for 3 weeks may be considered a T2DM rat model.

In recent years, mitochondrial biogenesis and its association with insulin resistance has gained interest. Mitochondrial biogenesis as a target of T2DM therapy has also been proposed and reviewed (10, 25). In the present study, the group representing type 2 diabetic conditions showed clear differences in mtDNA copy number under type 2 DM conditions. In particular, a marked 75% decrease in the soleus, whereas a 30% decrease was evident in the gastrocnemius muscle. This was in contrast to studies conducted using db/db diabetic mice in which almost no impairment of mitochondrial function in the gastrocnemius was observed (26). One possible reason for this is that mitochondria account for almost 6% of the soleus and less than 3% in gastrocnemius muscles (27). Zuo et al. (28) suggested that the soleus in individuals with T2DM is likely subject to tissue oxygenation stress, which may lead to a decrease in mtDNA copy number.

Wessels et al. (29) reported that treatment with metformin at 300 mg/kg/day impaired the in vivo and ex vivo skeletal muscle mitochondrial oxidative capacity in Zucker diabetic fatty rats. A previous study showed that metformin enhanced the PGC1α expression and mitochondrial biogenesis, possibly through AMPK phosphorylation in the skeletal muscle (30). In the present study, a 300 mg/kg/day dose of metformin ameliorated insulin sensitivity and significantly increased mtDNA copy number in soleus diabetic rats compared with controls (*P* < 0.05).

Analysis of mtDNA copy number in the gastrocnemius showed similar results between the metformin group and extract group at all doses, in which mtDNA copy number in the gastrocnemius increase by approximately 1.5 times compared with the diabetic group but did not significantly differ from the normal group. The activity of metformin and *T. diversifolia* extracts in the gastrocnemius muscle may only restore the mtDNA ratio to normal conditions. Moreover, the extract’s effect at increasing mtDNA copy number may be achieved at a starting dose of 100 mg/kg/day. At this dose, the mtDNA ratio in the soleus increased up to 3 times that of the normal group. Interestingly, the ability of the 100 mg/kg/day to increase the mtDNA ratio was greater compared with the 150 mg/kg/day dose (*P* < 0.001). Therefore, it is possible that increasing the dose of *T. diversifolia* extracts increases activity only to the optimal level. Chunudom et al. (31) also reported that *T. diversifolia* extracts at a dose of 100 mg/kg could increase GLUT2 expression in diabetic rat liver.

An in vitro study of *T. diversifolia* showed an influence on AMPK activation (8) and that it acts as a PPARα/γ agonist (7). Phosphorylated-AMPK is one pathway that activated during mitochondrial biogenesis (9). Therefore, studies are needed to identify the underlying mechanism of *T. diversifolia* in mitochondrial biogenesis, which in the present study, was shown to increase mtDNA copy number.

## Conclusion

A standardised extract of *T. diversifolia* showed anti-diabetic activity by improving insulin resistance and increasing mtDNA copy number, which suggests an improvement in mitochondrial biogenesis. However, further studies are needed to clarify the mechanism of *T. diversifolia* in improving insulin resistance as it relates to mitochondrial biogenesis.

## Figures and Tables

**Table 1:** Primer design

Gene	Accession number	Product size (bp)	Sequence (5’–3’)
β-actin	NC_005111.4	212	Forward: CCG CGA GTA CAA CCT TCT TG Reverse: CCT AGG CGG AAA GTT AAG CT
NADH dehydrogenase 1	NC_001665.2	181	Forward: AGC TCT AAG CCT ATG AAT CCC CT Reverse: AGA GAT GGT TTG GGC AAC GG

**Table 2:** Profile of standardised ethanol extract of *T. diversifolia*

Parameter	Results	Reference^1^
Specific parameter		
Qualitative analysis	Positive for flavonoid, alkaloid, saponin and polyphenol	
Tagitinin C identification	Rf: 0.56 (Rf standard: 0.56)	
Tagitinin C (% w/w)	7.60	
Non spesific parameter		
Water content (% v/w)	6.02	≤ 10
Total ash (% w/w)	11.67	-
Residual solvent	Negative	-
Microbial contamination		
• Plate total count (cfu/g)	< 10	≤ 10^4^
• Yeast and mold count (cfu/g)	< 10	≤ 10^3^
• *S. aureus*, *E. coli*, *Pseudomonas spp*., *Salmonella spp*.	Negative	Negative
Determination of heavy metals		
• Lead/Pb (mg/kg)	0.0185. 10^−3^	≤ 10
• Cadmium/Cd (mg/kg)	0.0004. 10^−3^	≤ 0.3

Notes:

1 According to the Indonesia National Agency of Drug and Food Control. No. 32 about requirements of safety and quality of traditional medicines (15)

**Table 3:** Profil of fasting blood glucose, fasting insulin and HOMA-IR score in baseline and after diabetes induction

	Groups						*F* test value	*P*-value

	Normal	Diabetic	Metformin	E50	E100	E150		
Baseline profile^1^								
Glucose (mmol/L)	5.82 (0.27)	5.53 (0.60)	5.73 (0.51)	5.88 (0.52)	6.13 (0.45)	5.90 (0.28)	0.971	0.455
Insulin (μU/mL)	2.26 (0.46)	2.76 (0.58)	2.57 (0.33)	2.44 (0.22)	2.96 (0.37)	1.99 (0.85)	2.300	0.077
HOMA-IR	0.88 (0.21)	1.01 (0.24)	0.97 (0.07)	0.95 (0.07)	1.20 (0.08)	0.77 (0.31)	1.571	0.206
Induction								
Glucose (mmol/L) 2	5.83 (0.55)	11.37 (2.74)	10.62 (1.84)	10.24 (1.20)	9.80 (0.91)	10.62 (1.64)	7.336	< 0.001*
*P*-value with normal group	–	0.0002*	0.001*	0.003*	0.009*	0.001*		
*P-*value with diabetic group		–	0.977	0.880	0.659	0.977		

Notes: Data are mean (SD) (*N* = 5). E50 is extract dose of 50 mg/kg group, E100 is extract dose of 100 mg/kg group and E150 is extract dose of 150 mg/kg group;

1 All data on baseline showed normal distribution and proceed to analysis of variance (ANOVA) test (DFn = 5, DFd = 24);

2 Glucose levels after induction showed normal distribution and it was continued with ANOVA test (DFn = 5, DFd = 24) followed by Tukey HSD multiple comparisons at 95% confidence interval (*)

**Table 4:** Profil of fasting blood glucose, fasting insulin and HOMA-IR score after 28 days treatment

	Groups						*F* test value

	Normal	Diabetic	Metformin	E50	E100	E150	
Glucose (mmol/L)^1^	6.22 (0.22)	11.65 (1.25)	6.11 (0.46)	6.03 (0.41)	6.18 (0.22)	5.54 (0.36)	74.304
*P*-value with normal group	–	< 0.001*	1.000	0.995	1.000	0.486	
*P*-value with diabetic group		–	< 0.001*	< 0.001*	< 0.001*	< 0.001*	
Insulin (μU/mL) 1	2.55 (0.55)	3.79 (0.49)	2.76 (0.45)	3.01 (0.35)	3.13 (0.42)	3.20 (0.30)	4.834
*P*-value with normal group	–	0.002*	0.976	0.572	0.331	0.212	
*P*-value with diabetic group		–	0.011*	0.082	0.185	0.293	
HOMA-IR^2^	1.05 (0.23)	2.93 (0.47)	1.11 (0.14)	1.20 (0.12)	1.28 (0.14)	1.18 (0.13)	–
*P*-value with normal group	–	0.009*	0.465	0.295	0.117	0.465	
*P*-value with diabetic group		–	0.009*	0.009*	0.009*	0.009*	

Notes: Data are mean (SD) (*N* = 5). E50 is extract dose of 50 mg/kg group, E100 is extract dose of 100 mg/kg group and E150 is extract dose of 150 mg/kg group;

1 Glucose levels and insulin level after 28 days treatment showed normal distribution and homogeneous, so it was continued with ANOVA test (DFn = 5, DFd = 24) followed by Tukey HSD multiple comparisons at 95% confidence interval (*);

2 HOMA-IR score showed non-parametric type, so it was continued with Mann-Whitney U test (2-tailed) and it was significantly different if *P* < 0.05 (*)

**Table 5:** Ratio of mtDNA in soleus and gastrocnemius after 28 days treatment

Ratio of mtDNA	Groups						*F* test value	*P*-value

	Normal	Diabetic	Metformin	E50	E100	E150		
Soleus	1.14 (0.56)	0.22 (0.03)	1.48 (0.21)	0.86 (0.35)	3.30 (0.39)	2.19 (0.29)	49.405	< 0.001*
*P*-value with normal group	–	0.004*	0.617	0.792	< 0.001*	< 0.001*		
*P*-value with diabetic group		–	< 0.001*	0.075	< 0.001*	< 0.001*		
Gastrocnemius	1.03 (0.30)	0.77 (0.15)	1.65 (0.54)	1.68 (0.61)	1.72 (0.49)	1.55 (0.15)	5.270	0.002*
*P*-value with normal group	–	0.771	0.216	0.174	0.131	0.395		
*P*-value with diabetic group		–	0.014*	0.010*	0.007*	0.033*		

Notes: Data are mean (SD) (*N* = 5). E50 is extract dose of 50 mg/kg group, E100 is extract dose of 100 mg/kg group and E150 is extract dose of 150 mg/kg group. Ratio of mtDNA in soleus and gastrocnemius muscles after 28 days treatment showed normal distribution and homogeneous, so it was continued with ANOVA test (DFn = 5, DFd = 24) followed by Tukey HSD multiple comparisons at 95% confidence interval and it was significantly different if *P* < 0.05 (*)
